# Extracellular zinc acts as a dynamic extracellular regulator of fertilization in *Xenopus laevis*^†^

**DOI:** 10.1093/biolre/ioag161

**Published:** 2026-07-31

**Authors:** Rachel E Bainbridge, Kayla M Komondor, Jennifer M Miller, Beatrice L Milnes, Olivia G Brado, Khaalis M T Harris, Paushaly Sau, Eirlys Barbara, Sarah Khanzada, Risha Musuku, Julia Schack, Anne E Carlson

**Affiliations:** Department of Biological Sciences, University of Pittsburgh, Pittsburgh, PA, United States; Department of Biological Sciences, University of Pittsburgh, Pittsburgh, PA, United States; Department of Biological Sciences, University of Pittsburgh, Pittsburgh, PA, United States; Department of Biological Sciences, University of Pittsburgh, Pittsburgh, PA, United States; Department of Biological Sciences, University of Pittsburgh, Pittsburgh, PA, United States; Department of Biological Sciences, University of Pittsburgh, Pittsburgh, PA, United States; Department of Biological Sciences, University of Pittsburgh, Pittsburgh, PA, United States; Department of Biological Sciences, University of Pittsburgh, Pittsburgh, PA, United States; Department of Biological Sciences, University of Pittsburgh, Pittsburgh, PA, United States; Department of Biological Sciences, University of Pittsburgh, Pittsburgh, PA, United States; Department of Biological Sciences, University of Pittsburgh, Pittsburgh, PA, United States; Department of Biological Sciences, University of Pittsburgh, Pittsburgh, PA, United States

**Keywords:** zinc, fertilization, polyspermy block, *Xenopus laevis,* egg activation, extracellular matrix, jelly coat, TMEM16A

## Abstract

For most animals, fertilization of an egg by more than one sperm is embryonically lethal, and eggs therefore deploy multiple blocks to prevent additional sperm from entering an already fertilized egg. While Zn^2+^ release has emerged as a conserved feature of the slow block, its precise role in external fertilizers remains unclear. Here, we demonstrate that *Xenopus laevis* fertilization triggers a robust efflux of Zn^2+^ and that insemination of *X. laevis* eggs in physiologically relevant concentrations of Zn^2+^ potently and reversibly suppresses early development. Our findings are consistent with the hypothesis that extracellular Zn^2+^ inhibits fertilization through multiple mechanisms. Extracellular Zn^2+^ prevents the fertilization-evoked depolarization, also called the fast block to polyspermy, by acting upstream of the Ca^2+^-activated Cl^−^ channel transmembrane protein 16A (TMEM16A). Zn^2+^ also directly reduces sperm fertilizing capacity, although eggs are significantly more sensitive than sperm. Furthermore, Zn^2+^ coordinates with the egg’s extracellular matrix to stabilize the glycoprotein-rich jelly coat, rendering it resistant to reductive removal and suggesting a structural mechanism by which Zn^2+^ limits sperm entry. The inhibitory effects of Zn^2+^ are fully reversed by extracellular Zn^2+^ chelator (ZX1), reinforcing the conclusion that Zn^2+^ functions as a dynamic extracellular regulator rather than an intracellular messenger. Our findings establish Zn^2+^ as a multi-level signal that reinforces fertilization barriers by targeting both gametes and modifying the egg’s extracellular environment.

## Teaser

Extracellular Zn^2+^ released at fertilization acts on both gametes and the egg's extracellular matrix to reversibly suppress fertilization in Xenopus laevis.

## Graphical Abstract

**Graphical Abstract:**
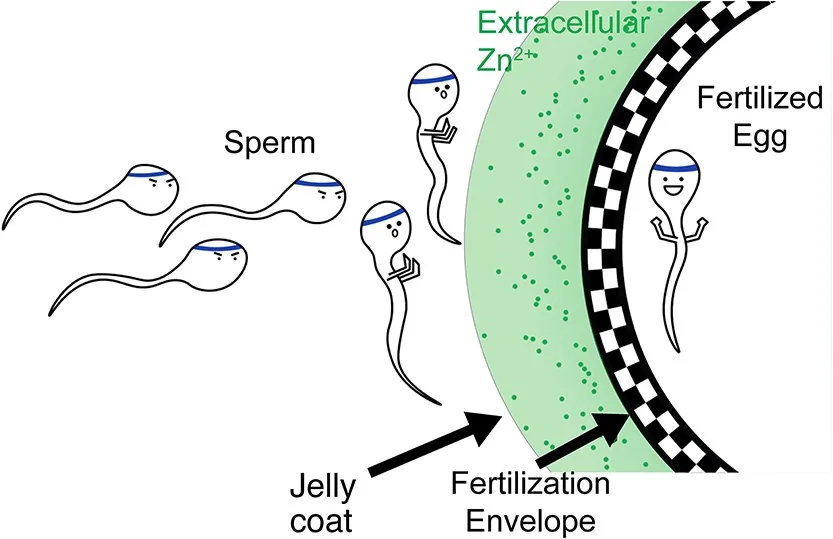

## Introduction

Polyspermy, the fertilization of an egg by more than one sperm, is embryonically lethal for most animals. To prevent this, eggs have evolved robust mechanisms that block additional sperm entry [1]. One of these, the fast block to polyspermy, involves a fertilization-triggered depolarization of the egg membrane [2]. It occurs within seconds of sperm entry and is only observed in animals with external fertilization [3, 4]. By contrast, the slow block develops over minutes and is present in eggs from nearly all animals [5].

The slow block is initiated by the fertilization-triggered exocytosis of cortical granules from the eggs into the extracellular environment [6]. Although the complete molecular composition of these granules remains incompletely characterized, their contents are known to remodel the egg membrane and its surrounding extracellular matrix, thereby reducing sperm binding and penetration [7–10]. Among the components released during this process is Zn^2+^, a metal ion increasingly recognized as an important regulator of fertilization-associated events [11].

Recent studies have demonstrated that eggs from diverse species, including humans, mice, cows, frogs, salamanders, fish, worms, and flies, release Zn^2+^ into the extracellular space at fertilization [12–18]. This extracellular Zn^2+^ is proposed to modify egg-associated structures in a manner consistent with a supporting role in the slow block to polyspermy [18–20]; however, proposed mechanisms, including effects on sperm motility and zona pellucida remodeling, remain incompletely defined. Moreover, chelation or genetic depletion of egg zinc transporters does not increase polyspermy rates [21, 22], indicating that Zn^2+^ is not required for polyspermy prevention per se but may instead act as a modulatory signal that reinforces fertilization barriers through actions on both gametes and the egg’s extracellular environment. In the African clawed frog, *Xenopus laevis*, fertilization elicits a single, coordinated wave of Zn^2+^ release that coincides with cortical granule exocytosis and lifting of the fertilization envelope, two hallmark features of the slow block [17, 18]. Exposure of *X. laevis* eggs to physiologically relevant concentrations of extracellular Zn^2+^ suppresses fertilization and early embryonic development [18], consistent with extracellular Zn^2+^ release playing a broadly conserved modulatory role in fertilization across taxa [11].

Despite this emerging appreciation of Zn^2+^ as a fertilization-associated signal, the mechanisms by which extracellular Zn^2+^ suppresses fertilization remain only partially defined, particularly in externally fertilizing species. Although recent work has begun to elucidate the sources of Zn^2+^ release and its role in zona pellucida remodeling and sperm–egg interaction in mammals [20–22], the cellular targets of Zn^2+^ and the mechanisms by which it influences fertilization-associated events, as well as the manner in which Zn^2+^ interacts with the egg’s extracellular structures, remain incompletely characterized.

Here, we investigate how extracellular Zn^2+^ regulates fertilization in *X. laevis*. Building on our previous demonstration that fertilization triggers Zn^2+^ release during the temporal window of the slow block [18], we define the physiological consequences of extracellular Zn^2+^ exposure and identify multiple cellular and extracellular mechanisms through which Zn^2+^ suppresses fertilization-associated events. Our findings reveal extracellular Zn^2+^ as a dynamic regulator of fertilization that acts on both gametes, modifies the egg’s extracellular environment, and operates through reversible, extracellular mechanisms, thereby uncovering a previously unrecognized layer of ionic control in the earliest stages of embryonic development.

## Methods

### Ethics

All experimental procedures involving vertebrate animals were conducted in accordance with the National Institutes of Health Guide for the Care and Use of Laboratory Animals and were approved by the Institutional Animal Care and Use Committee at the University of Pittsburgh.

### Animals

Captively bred *X. laevis* adults were purchased from *Xenopus* 1 (Dexter, MI) and housed at 18°C with a 12/12-h light/dark cycle.

### Reagents

Extracellular Zn^2+^ chelator (ZX1) was purchased from Strem Chemical (Newburyport, MA) and dispensed from a 100 mM stock solution prepared in dimethyl sulfoxide (DMSO), and human chorionic gonadotropin (hCG) was purchased from Covestrus (Melville, NY). A 0.1 M ZnSO_4_ solution was purchased from Thermo Fisher and used to make all solutions. Unless otherwise noted, all other reagents were purchased from Thermo Fisher Scientific.

### Solutions

Modified Ringers (MR) solution was used for *X. laevis* fertilization experiments. MR contains (in mM): 100 NaCl, 1.8 KCl, 2.0 CaCl_2_, 1.0 MgCl_2_, and 5.0 HEPES, pH 7.8, and is filtered using a sterile, 0.2 μm polystyrene filter [23]. Embryonic development assays were performed in 33% MR diluted in DDH_2_O (MR/3).

Oocyte Ringers 2 (OR2) solution was used to rinse *X. laevis* oocytes after collagenase treatment. OR2 is comprised of (in mM): 82.5 NaCl, 2.5 KCl, 1 MgCl_2_, and 5 mM HEPES, pH 7.6, and is filtered using a sterile, 0.2 μm polystyrene filter [24]. The following solution was used to store *X. laevis* oocytes (in mM): 96 NaCl, 2 KCl, 1 MgCl_2_, 10 HEPES, 5 sodium pyruvate, and 10 mg/L gentamycin at pH 7.6 and is filtered with a sterile 0.2 μm polystyrene filter [25].

### Collection of *Xenopus laevis* gametes

Eggs were collected from sexually mature females by procedures described previously [26, 27]. Egg-laying was stimulated by the injection of 1000 IU of hCG dissolved in water (1000 IU per 100 μL) into the dorsal lymph sac. Following injection, females were housed overnight for 12–16 h at 14–16°C. Typically, egg-laying began within 2 h of the females being moved to room temperature. Eggs were collected on dry petri dishes and used within 10 min of being laid.

Sperm were obtained from testes harvested from sexually mature *X. laevis* males [23]. Following euthanasia by a 30-min immersion in 3.6 g/L tricaine-S (pH 7.4), testes were dissected and cleaned by manual removal of residual fat and vasculature. Unlike mammalian sperm preparations, which are typically obtained from the cauda epididymis or vas deferens as a quantifiable cell suspension, *X. laevis* sperm are prepared directly from testicular tissue. Cleaned testes were stored at 4°C in MR for use on the day of dissection or in L-15 medium for up to 1 week. Sperm were extracted by mincing 1/10 of a testis in 200–500 μL of MR and were used within 1 h of collection [23].

Immature oocytes were collected from *X. laevis* females anesthetized by immersion in 1.0 g/L tricaine (pH 7.4) for 30 min [28]. Ovarian sacs that included developing oocytes were removed from the female, manually separated, and incubated for 90 min in 1 mg/mL collagenase diluted in a modified ND96 solution lacking pyruvic acid and gentamycin. Several rinses removed collagenase in the OR2 solution, and healthy oocytes were stored at 14°C in ND96 for up to 14 days [29].

### Quantification of released Zn^2+^

To quantify extracellular Zn^2+^ released during *X. laevis* fertilization, jelly was removed from batches of 22–158 freshly laid eggs. Jelly-free eggs were inseminated with *X. laevis* sperm in a known volume of MR/3 solution with 5 mM HEPES in a glass dish. The solution surrounding the eggs was collected 20–45 min after sperm addition, when visible signs of activation were noted. Exudate solution was collected and transferred using glass Pasteur pipettes. Solution was sedimented at 3000 rpm for 5 min to pellet sperm, and the supernatant was transferred to a new tube (*N* = 8, 22–158 eggs, 6 frogs).

FluoZin-3 tetrapotassium salt was dispensed from a 1 mM stock solution in water, diluted into a 500 nM working stock, then diluted into standards or experimental solutions for a final concentration of 50 nM. Post-fertilization exudate was diluted 1:1100 in purified water prior to measurement; multiple dilutions were assessed for each sample to ensure linearity of the signal within the standard curve range. To account for zinc contamination in the assay buffer, raw photometric signals were corrected by subtracting the FluoZin-3-free background signal, which was collected before adding the fluorophore to each sample. In the case of standards, these corrected signals were additionally corrected by subtracting the background from solution containing N,N,N′,N′-tetrakis(2-pyridinylmethyl)-1,2-ethanediamine (TPEN). Exudate samples were additionally corrected by subtracting the background fluorescence of MR/3 solution. Fluorescence intensity measurements were recorded in a TECAN SPARK fluorescence plate reader (Tecan, Männedorf, Switzerland). FluoZin-3-containing samples were excited at 494 nm (7.5 nm bandwidth), and emission was collected using the instrument’s 516 nm (7.5 nm bandwidth) emission channel, which overlaps with the emission maximum of Zn^2+^-bound FluoZin-3. The Zn^2+^ was quantified using a standard curve calculated with the hill equation [30]:


$$ \left[{Zn}^{2+}\right](nM)=\frac{X_{half}}{{\left(\frac{\left(\max - base\right)}{\left( RFU- base\right)}-1\right)}^{\frac{1}{rate}}} $$

where *RFU* is the measured fluorescence intensity of each sample, base is the empirically measured background fluorescence, max is the maximum fluorescence at Zn^2+^ saturation, x_half_ is the half-maximal Zn^2+^ concentration derived from the standard curve fit, and rate is the Hill coefficient, fixed at 1 to reflect the 1:1 binding stoichiometry of Zn^2+^ to FluoZin-3. The constants base, max, and x_half_ were determined empirically for each experimental trial from ZnCl_2_ standards buffered with 100 μM Nitrilotriacetic acid (NTA) in MR/3. ZnCl_2_ standard concentrations range from 3.5 to 99.3 μM for labile Zn^2+^ concentrations of 0.1–100 nM. Free Zn^2+^ concentrations were calculated using MaxChelator (WEBMAXC STANDARD) [31]. Background fluorescence was assessed with NTA buffered MR/3 with added 50 μM TPEN. Minimum fluorescence was based on NTA-buffered MR/3 with no added ZnCl_2_, and max fluorescence with 50 μM ZnCl_2_.

To determine the number of Zn^2+^ ions released per egg, the total Zn^2+^ calculated concentration was multiplied by the dilution factor and Avogadro’s number, then divided by the volume of solution in which eggs were inseminated and the number of eggs per trial. Averages of calculated Zn^2+^ ion counts are reported with standard error.

### Pre-treatment before insemination

For experiments in which eggs were sequentially treated under different conditions, the eggs were incubated in an initial experimental solution, washed three times by transferring them between petri dishes containing the final treatment solution using a plastic transfer pipette to remove the initial treatment, and then placed in the final treatment solution. For experiments pre-treating sperm with different conditions, 1/10 of a testis was minced in 200 μL of the pretreatment solution, then allowed to incubate on ice for 15 min. For insemination, 20 μL of the sperm suspension was added to eggs bathed in 3 mL of the final solution. The specific solutions used for pre-treatment and insemination in each experiment, including their Zn^2+^ or ZX1 concentrations, are described in the Results section for each individual experiment and correspond to the conditions depicted in the relevant figures.

### *Xenopus laevis* egg imaging and development assay

Inseminated eggs and developing embryos were imaged using an Edmund Optics stereomicroscope with a 10× objective, fitted with a pixiLINK digital camera and the μScope Essential 64× software (pixiLINK, Canada).

For each experimental trial, the development of *X. laevis* embryos was assessed from approximately 20–40 eggs in each experimental condition. 20–90 μL of the sperm suspension was used to fertilize eggs, depending on the volume of the dish. Approximately 90–120 min after insemination, the appearance of cleavage furrows was used to assess the initiation of embryonic development [23]. Each assay was repeated at least three times using gametes from different males and females, and on different experimental days. Percentages of development from these assays were reported as the average with standard error. In instances of concentration responses, a sigmoidal fit of development incidence versus metal ion concentration yielded a half-maximal inhibitory concentration, which was averaged and reported with standard error. In the cases where dose responses were compared, a one-way analysis of variance (ANOVA) with Tukey’s honest significant difference (HSD) post hoc test was used to determine statistical differences.

### Electrophysiology

Whole-cell recordings on *X. laevis* eggs during fertilization [32] were made using TEV-200A amplifiers (Dagan Co.) and digitized by Axon Digidata 1550A (Molecular Devices). Data were acquired using pClamp Software (Molecular Devices) at 5 kHz. Pipettes used to impale the *X. laevis* eggs for recordings were pulled from borosilicate glass for a resistance of 5–15 MW and filled with 1 M KCl.

In whole-cell recordings, resting and fertilization potentials were quantified ~10 s before and after depolarization, respectively. Depolarization rates of each recording were quantified by determining the maximum velocity of the quickest 1-mV shift in the membrane potential [26]. These rates, resting potentials, and depolarization times were averaged and reported with standard error.

For two-electrode voltage-clamp recordings, *X. laevis* oocytes were clamped at −80 mV to record inositol trisphosphate (IP_3_)-evoked currents. Each oocyte was injected with a 200 μM caged IP_3_ stock in DDH_2_O to achieve a final concentration of 5 μM within the oocyte and incubated for at least 30 min in the dark at 18°C before recording [33]. IP_3_ was released from the nitrophenyl cage by flash photolysis via 250-ms ultraviolet (UV) light exposure (Ultra High Power White LED Illuminator, 380–603 nm; Prizmatix). The bath solution in our recording chamber (RC-26G; Warner Instruments) was changed using the gravity-fed pinch valve solution changer (VC-8; Warner Instruments).

### Egg jelly removal

For procedures requiring the removal of jelly, eggs were incubated at room temperature for 5 min before being inseminated with a sperm suspension prepared as previously described [18]. To remove the jelly, eggs were placed on agar in a 35 mm petri dish in MR/3 medium with 45 mM β-mercaptoethanol (βME), pH 8.5. During the βME incubation, eggs were gently agitated for 1–2 min, until their external jelly visibly dissolved, as indicated by close nestling of the eggs and loss of visible jelly. To remove the βME solution, de-jellied eggs were then moved with a plastic transfer pipette to a petri dish coated with 1% agar in MR/3 pH 6.5 and agitated for an additional minute. Eggs were then transferred three times to additional agar-coated dishes with MR/3 pH 7.8, swirling gently and briefly in each.

### Statistical analysis

Details for each statistical method used, including specific tests applied, are described in the Results section.

## Results

### Extracellular Zn^2+^ functionally inhibits early development in a dose-dependent manner

To explore how Zn^2+^ may contribute to fertilization and early embryonic development in *X. laevis*, we quantified Zn^2+^ release into the extracellular medium during the slow block to polyspermy [18]. We detected a robust and reproducible release of Zn^2+^ within minutes of fertilization, corresponding to an average of 1.2 × 10^13^ Zn^2+^ ions released per egg (*N* = 8 samples from 6 independent experiments, from clutches of 22–158 eggs; Supplemental Figure S1). These values closely match previous measurements from our laboratory using both fluorimetric assays and inductively coupled plasma mass spectrometry [18], and demonstrate that fertilization triggers a robust efflux of Zn^2+^ into the surrounding environment, with a geometric mean release of 8.880 pmol/egg (geometric SD factor 4.8, *N* = 8 independent experiments; Supplemental Figure S1B). Together, these findings establish that Zn^2+^ temporally and spatially influences subsequent fertilization events.

Motivated by this temporal and spatial positioning of Zn^2+^, we reasoned that if extracellular Zn^2+^ protects the nascent zygote from additional sperm entry, eggs inseminated in low Zn^2+^ concentrations should develop normally. To test this, we inseminated *X. laevis* eggs in varying concentrations of extracellular Zn^2+^ and assessed cleavage furrow formation (Supplemental Figure S2A), a hallmark of early embryonic development. Increasing extracellular Zn^2+^ progressively inhibited cleavage furrow formation (Supplemental Figure S2B), and no development occurred in eggs inseminated with ≥300 μM ZnSO_4_. A sigmoidal fit of development incidence versus ZnSO_4_ concentration yielded a half-maximal inhibitory concentration (IC_50_) of 24 ± 4 μM (*N* = 74–97 eggs in 5 trials; Supplemental Figure S2B). This is consistent with previous reports from our lab (30 ± 10 μM [18]) and from others (53 μM, 95% confidence interval = 47–61 μM [17]). These observations prompted us to examine whether extracellular Zn^2+^ interferes with the earliest physiological hallmark of fertilization-associated events.

### Extracellular Zn^2+^ prevents the fast block to polyspermy

To explore whether extracellular Zn^2+^ interferes with fertilization-associated events, we monitored the earliest indicator of fertilization in *X. laevis*, the fast block to polyspermy [2]. We performed whole-cell recordings on *X. laevis* eggs before and after sperm addition, in the presence or absence of Zn^2+^ (Figure 1A–E). Under control conditions, eggs exhibited an average resting potential of −11.0 ± 2.5 mV, and sperm addition triggered a rapid depolarization to 17.9 ± 4.7 mV (*N* = 7) (Figure 1B and D). This depolarization occurred 6.8 ± 0.8 min after sperm addition and proceeded at a rate of 0.66 ± 0.45 mV/ms (Figure 1E).

**Figure 1:**
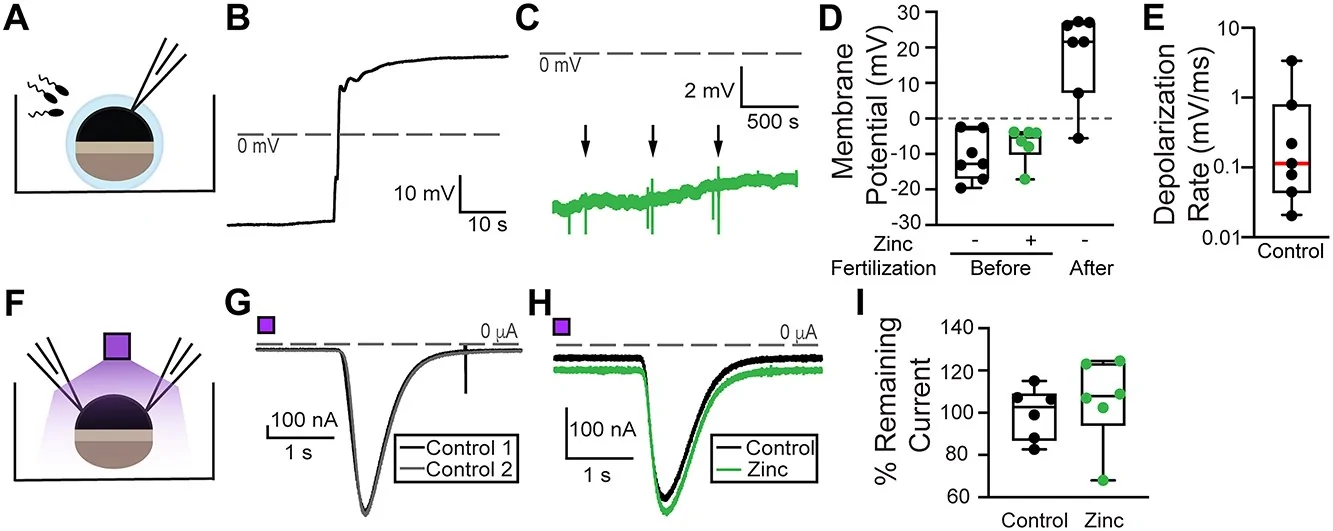
Extracellular Zn^2+^ abolishes the fertilization-evoked depolarization without inhibiting TMEM16A. (A) Schematic of whole-cell recordings made from *X. laevis* eggs during fertilization. (B and C) Representative whole-cell membrane potential recordings during fertilization under control conditions (B) or in 300 μM Zn^2+^ (C). The dashed line indicates 0 mV. In panel C, black arrows denote sperm additions. (D) Box plot distribution of membrane potentials recorded from eggs before fertilization under control solutions or with 300 μM Zn^2+^, and after fertilization under control conditions only, as only control eggs underwent a fertilization-evoked depolarization. (E) Distribution of depolarization rates recorded under control conditions (no Zn^2+^). (F) Schematic of two-electrode voltage clamp recordings from *X. laevis* oocytes injected with a photolabile IP_3_ analog, clamped at −80 mV. Shown are typical currents before and after a 250-ms UV uncaging pulse in oocytes in control solutions (G) or in 300 μM Zn^2+^ (H). (I) Box plot distribution of relative peak current remaining after successive uncaging events, shown under control conditions and in 300 μM Zn^2+^.

By contrast, eggs inseminated in 300 μM ZnSO_4_ did not depolarize (Figure 1C, *N* = 6). To ensure that the absence of a depolarization was not due to insufficient sperm exposure, sperm were added three times at 10-min intervals over at least 30 min. Zn^2+^-treated eggs maintained a stable resting potential of −7.2 ± 2.0 mV (Figure 1D) and never exhibited signs of embryonic development, such as contraction of the animal pole or the appearance of cleavage furrows. Together, these observations indicate that extracellular Zn^2+^ profoundly disrupts the early events of fertilization.

One possible explanation for the absence of depolarization is that Zn^2+^ could enter the egg and inhibit the Ca^2+^-activated Cl^−^ channel, transmembrane protein 16A (TMEM16A), the channel responsible for the fertilization-evoked depolarization [26]. Zn^2+^ is known to compete with Ca^2+^ for binding to TMEM16A’s calcium-binding site when applied to the intracellular surface of excised patches, although it does not activate the channel [34]. This raises the possibility that intracellular Zn^2+^ competitively suppresses Ca^2+^-dependent activation of TMEM16A. To test this directly, we performed two-electrode voltage clamp experiments on *X. laevis* oocytes loaded with photolabile IP_3_ [26, 29]. A 250-ms pulse of UV light uncages IP_3_, triggering Ca^2+^ release and TMEM16A activation.

Under control conditions, successive applications of UV light to uncage IP_3_ produced currents of comparable amplitude, with the second uncaging yielding an average of 100 ± 5% of the first response (*N* = 6, Figure 1G and I). After a 10-min incubation in 300 μM ZnSO_4_, the second uncaging evoked a similar current (105 ± 8%, *N* = 6, Figure 1H and I, *P* = 0.55, *t-*test). These results indicate that Zn^2+^ does not interfere with IP_3_-signaled, Ca^2+^-dependent activation of TMEM16A. Accordingly, the absence of fertilization-evoked depolarization in Zn^2+^-treated eggs suggests that Zn^2+^ acts upstream of the egg’s electrical response and is consistent with inhibition of fertilization-associated events. We therefore examined whether this inhibitory effect reflects direct actions of Zn^2+^ on the egg or sperm.

### Extracellular Zn^2+^ inhibits fertilization by targeting both gametes

Because fertilization requires the coordinated function of both gametes, we tested whether extracellular Zn^2+^ acts on the egg, the sperm, or both. To test whether Zn^2+^ modifies the egg, we treated *X. laevis* eggs with increasing concentrations of extracellular Zn^2+^, washed extensively in our HEPES-buffered saline solution, and then inseminated under control conditions. We assessed fertilization by scoring cleavage furrow formation [23]. Zn^2+^ pretreatment of eggs inhibited fertilization, even when insemination occurred in control conditions. A sigmoidal fit yielded an IC_50_ of 70 ± 9 μM (*N* = 93–255 eggs, 6 trials; Figure 2A), not significantly different from the IC_50_ determined from the sigmoidal fit in Supplemental Figure S2B when insemination occurred in Zn^2+^-containing solution (Supplemental Figure S2B, *P* = 0.94, one-way ANOVA with Tukey’s HSD post hoc test).

**Figure 2:**
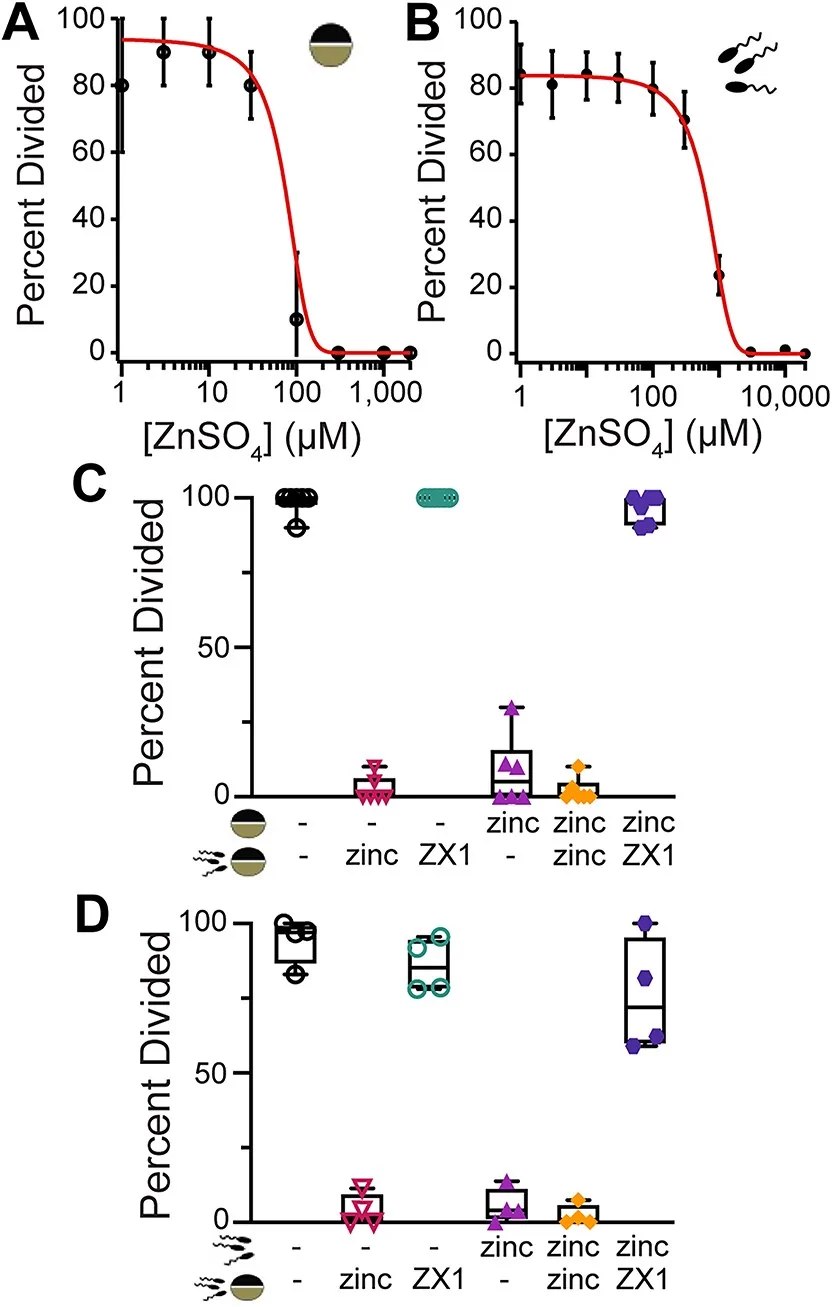
Extracellular Zn^2+^ reversibly suppresses fertilization-associated events through actions on both gametes and is rescued by chelation. (A) Eggs pretreated with increasing concentrations of extracellular Zn^2+^ exhibit a progressive reduction in the percentage of embryos undergoing early development during in vitro fertilization assays (*N* = 93–255 eggs, 6 independent trials). (B) The percentage of eggs undergoing embryonic development similarly decreases when inseminated with sperm pretreated with increasing concentrations of extracellular Zn^2+^ (*N* = 152–355 eggs, 5 independent trials). Data in A and B were fit with sigmoidal functions. (C and D) Box plots showing the percentage of eggs undergoing embryonic development following pretreatment of eggs or sperm under the following conditions: in (C), eggs were pretreated with 0 or 1 mM Zn^2+^, washed, and inseminated in solutions containing 0 or 1 mM Zn^2+^ or 300 μM ZX1 (*N* = 106–200 eggs, 6 independent trials). In (D), sperm were pretreated with 0 or 1 mM Zn^2+^ and used to inseminate eggs in solutions containing 0 or 1 mM Zn^2+^ or 300 μM ZX1 (*N* = 182–334 eggs, 4 independent trials).

To determine whether Zn^2+^ also targets sperm, sperm were pretreated in increasing Zn^2+^ concentrations for 15 min and used to inseminate eggs in a Zn^2+^-free solution. Fertilization decreased in a concentration-dependent manner (IC_50_ of 524 ± 102 μM, *N* = 152–355 eggs, 5 trials; Figure 2B), consistent with Zn^2+^ suppressing fertilization-associated events through both gametes, although eggs are significantly more sensitive than sperm (IC_50(egg)_ = 70 μM vs IC_50(sperm)_ = 524 μM, *P* = 0.04, one-way ANOVA with Tukey’s HSD post hoc test).

We next explored whether Zn^2+^’s inhibitory effect is reversible. Because ZX1 is a membrane-impermeant, extracellular Zn^2+^ chelator [35], we used it to directly test whether the inhibitory effects of Zn^2+^ depend on extracellular Zn^2+^. When eggs were pretreated with 1 mM ZnSO_4_, washed, and then inseminated in the presence of 300 μM ZX1, 100% developed cleavage furrows, indistinguishable from control eggs that had not been exposed to Zn^2+^ (95 ± 2%; Figure 2C). Moreover, ZX1 alone had no effect on development in eggs not previously exposed to Zn^2+^ (98 ± 2%; Figure 2C). Similarly, only 5.4 ± 2.5% of eggs developed cleavage furrows when inseminated with sperm pretreated in 1 mM ZnSO_4_ (*N* = 182–334 eggs, 4 trials; Figure 2D), whereas 75.8 ± 8.2% developed cleavage furrows when insemination occurred in the presence of ZX1 (Figure 2D). Importantly, the rate of development obtained with Zn^2+^-pretreated sperm inseminated in ZX1 did not differ significantly from control conditions (one-way ANOVA with Tukey’s post hoc test, *P* = 0.1297). Thus, extracellular chelation by ZX1 fully rescues fertilization in both Zn^2+^-pretreated eggs and sperm, without affecting fertilization under control conditions.

Together, these results demonstrate that extracellular Zn^2+^ potently but reversibly inhibits fertilization by targeting both gametes, with eggs displaying markedly greater sensitivity. Given the heightened sensitivity of the egg to Zn^2+^, we next explored whether Zn^2+^ alters the egg’s extracellular structures that regulate sperm entry.

### Extracellular Zn^2+^ stabilizes the *Xenopus laevis* jelly coat

*Xenopus laevis* eggs are surrounded by a glycoprotein-rich jelly coat that sperm must penetrate before reaching the egg [36, 37]. Disulfide bonds contribute to the structural integrity of this jelly matrix, as treatment with reducing agents such as βME can soften or remove the coat. Because Zn^2+^ can coordinate free cysteine thiol and histidine imidazole groups within glycoprotein matrices, we hypothesized that extracellular Zn^2+^ reinforces the structural integrity of the jelly coat through non-covalent coordination of amino acid residues within the glycoprotein-rich jelly matrix. To test this, we examined the ability of βME to remove the jelly coat in eggs following incubation with or without Zn^2+^. Jelly removal was assessed by measuring egg diameter before and after βME treatment (Figure 3A and B), with the rationale that loss of the jelly layer decreases the apparent diameter of the egg proper.

**Figure 3:**
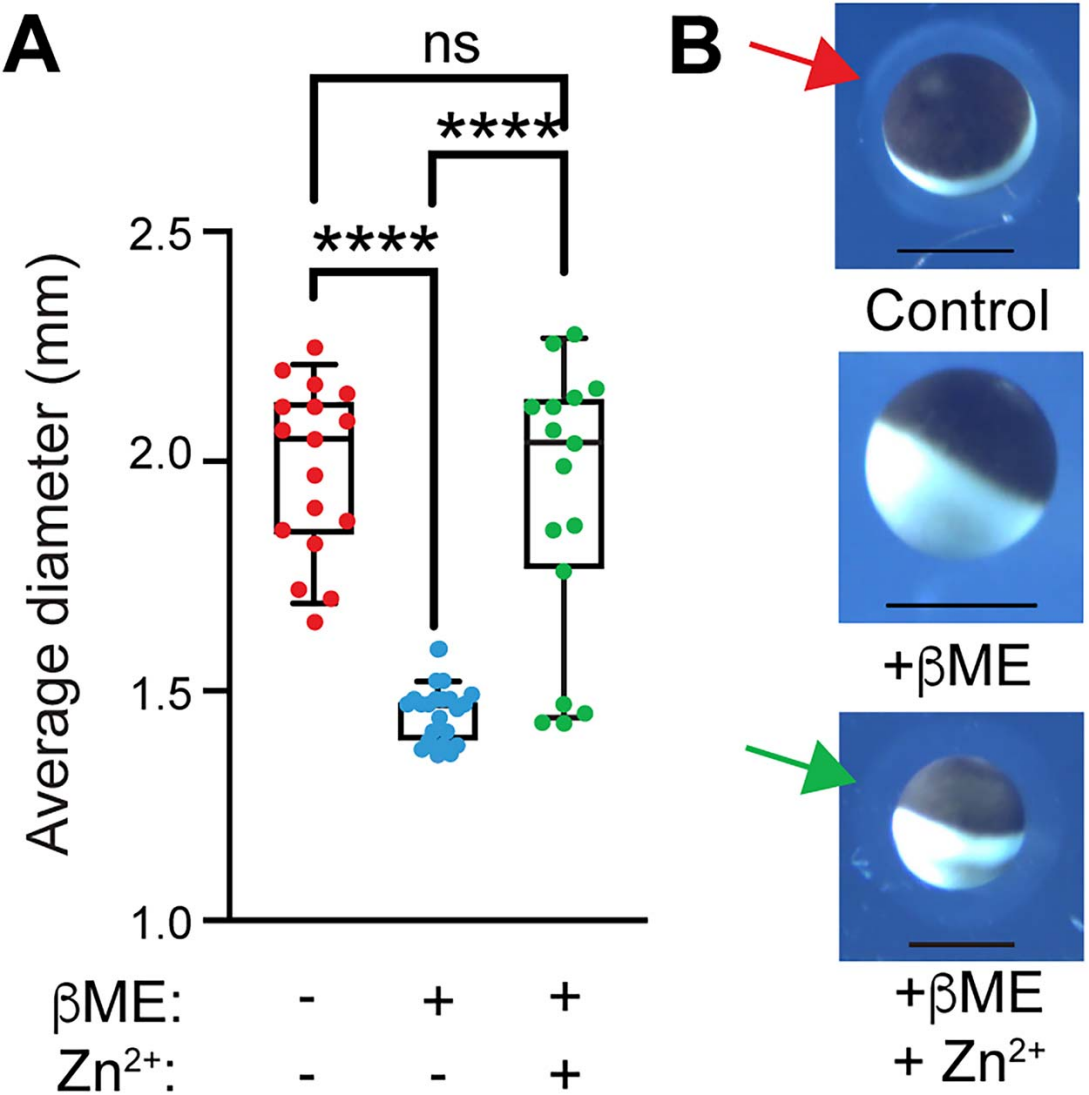
Extracellular Zn^2+^ stabilizes the *X. laevis* jelly coat. (A) Distribution of egg diameters measured under the same conditions: untreated control, βME alone, and Zn^2+^-pretreated eggs followed by βME. (B) Images of *X. laevis* eggs under three conditions: untreated control eggs (top), eggs treated with 300 μM Zn^2+^ before βME application (middle), and eggs following βME treatment to remove the jelly coat (bottom). Arrows indicate the edge of the jelly coat. Scale bars, 1 mm. Statistical significance was determined by ordinary one-way ANOVA with Tukey’s multiple comparisons test; (****) denotes *P* < 0.0001.

Under control conditions, egg diameter decreased from 1.98 ± 0.05 mm to 1.45 ± 0.01 mm after βME treatment (Figure 3, *N* = 17–22 eggs, *P* < 0.0001, one-way ANOVA with Tukey’s HSD post hoc test). By contrast, eggs preincubated in 1 mM Zn^2+^ showed no significant reduction in diameter (1.93 ± 0.07 mm, *N* = 15 eggs) and did not differ significantly from untreated eggs (*P* = 0.7440).

Together, these results indicate that exposure to extracellular Zn^2+^ renders the jelly coat resistant to reductive softening, consistent with enhanced structural stabilization of the jelly matrix in the presence of Zn^2+^.

## Discussion

In previous work, we demonstrated that fertilization in *X. laevis* is accompanied by a rapid extracellular release of Zn^2+^ during the temporal window of the slow block to polyspermy and that extracellular Zn^2+^ reversibly suppresses fertilization and early embryonic development across species [18]. Consistent with these observations, Zn^2+^ has emerged as a broadly conserved regulator of fertilization across taxa [11]. In the present study, we define how extracellular Zn^2+^ modulates fertilization-associated events in *X. laevis* and identify multiple cellular and extracellular mechanisms through which Zn^2+^ exerts its effects. Although Zn^2+^ release coincides with the slow block window, our data indicate that Zn^2+^ does not function as a classical polyspermy block component. This view is strongly supported by recent genetic studies in mice, which demonstrate that loss of fertilization-evoked Zn^2+^ release does not increase polyspermy, despite clear effects on zona pellucida remodeling and fertilization dynamics [21]. Instead, Zn^2+^ acts as a modulatory signal that reinforces fertilization barriers and shapes the egg’s extracellular environment following fertilization.

Our results indicate that extracellular Zn^2+^ suppresses fertilization-associated events through coordinated actions on both gametes and on the egg’s extracellular matrix. Zn^2+^ does not interfere with the egg’s intracellular activation machinery, as Ca^2+^-dependent activation of TMEM16A remains intact. Instead, Zn^2+^ acts upstream of the egg’s electrical response, preventing the physiological events that typically initiate fertilization-evoked depolarization. In parallel, Zn^2+^ directly reduces sperm fertilizing capacity in a concentration-dependent manner, although eggs are significantly more sensitive than sperm, suggesting that the egg is the primary target at physiologically relevant Zn^2+^ concentrations.

In addition to its effects on gametes, Zn^2+^ modifies the egg’s extracellular environment. We show that Zn^2+^ stabilizes the glycoprotein-rich jelly coat, rendering it resistant to reductive removal. We propose that this stabilization occurs through the ability of Zn^2+^ to coordinate free cysteine thiol and histidine imidazole groups within the glycoprotein matrix, both of which are well-established zinc ligands in biological systems. In this model, Zn^2+^ coordination of free residues would not require disruption of existing disulfide bonds; rather, non-covalent coordination complexes may physically impede access of βME to disulfide bonds, thereby rendering the jelly coat resistant to reductive removal. We acknowledge that we have not directly demonstrated that βME reduces disulfide bonds in the jelly coat in the presence or absence of Zn^2+^, and therefore interpret this result cautiously as evidence that Zn^2+^ alters the physical properties of the jelly coat in a manner consistent with the coordination of amino acid residues within the glycoprotein matrix. Because the jelly coat constitutes the first physical barrier encountered by sperm [38–40], this Zn^2+^-dependent alteration of jelly coat properties may restrict sperm penetration and contribute to the reduction in fertilization competence we observe. In this model, Zn^2+^-mediated stabilization of the jelly coat would prevent sperm from reaching the egg plasma membrane entirely, thereby blocking the initial sperm-egg interaction that normally triggers Ca^2+^ release and downstream TMEM16A activation.

We note that we have not directly tested whether chelation of Zn^2+^ with ZX1 restores the jelly coat’s sensitivity to reductive removal and therefore cannot conclude whether this effect is reversible. However, the broader inhibitory effects of Zn^2+^ on fertilization competence in both eggs and sperm are fully reversible upon chelation with ZX1, indicating that Zn^2+^ does not permanently alter gamete function. Although a precise local Zn^2+^ concentration within the jelly coat cannot be determined, as it remains unknown whether released Zn^2+^ distributes uniformly throughout the jelly volume or is retained preferentially in the layers closest to the egg, and what proportion binds to the vitelline envelope, we note that cortical granule exocytosis occurs at the egg surface, releasing Zn^2+^ directly into the jelly microenvironment. This spatial proximity suggests that local Zn^2+^ concentrations within the jelly are likely to substantially exceed bulk solution estimates. Given that the IC_50_ for fertilization inhibition is 24 ± 4 μM, and that a geometric mean of 8.8 pmol/egg (geometric SD factor 4.8, *N* = 8) is released at fertilization into this restricted microenvironment, the amount of Zn^2+^ released is plausibly sufficient to reach inhibitory concentrations within the jelly coat and contribute to the reduction in fertilization competence we observe. Together, these considerations support the plausibility of Zn^2+^-mediated jelly coat stabilization as a physiologically relevant mechanism, even in the absence of a precise local concentration estimate.

A defining feature of Zn^2+^-mediated regulation is its reversibility. Chelation of extracellular Zn^2+^ rapidly restores fertilization competence in both gametes, demonstrating that Zn^2+^ functions as a dynamic extracellular regulator rather than as a permanent structural block. The sufficiency of the membrane-impermeant chelator ZX1 further establishes the extracellular space as the principal site of Zn^2+^ action, reinforcing the conclusion that Zn^2+^ acts as an extracellular modulator of fertilization rather than an intracellular second messenger [35]. We acknowledge that the membrane impermeability of ZX1 rests on its sulfonate moiety conferring a negative charge at physiological pH, and that protonation of its pyridine and ethylenediamine nitrogen could in principle generate a neutral zwitterion capable of membrane permeation under some conditions [41]. However, several lines of functional evidence argue against ZX1 acting intracellularly under our experimental conditions. ZX1 alone does not activate eggs parthenogenetically, does not alter the membrane potential of unfertilized eggs in our electrophysiology experiments, and has no effect on fertilization rates in the absence of Zn^2+^ pretreatment. Furthermore, the rate of abnormal cleavage furrow formation in eggs fertilized in the presence of ZX1 (10%) is comparable to that observed under control conditions (15%), inconsistent with parthenogenetic activation. Together, these observations support the conclusion that ZX1 acts predominantly in the extracellular space under our experimental conditions, consistent with its established use as an extracellular Zn^2+^ chelator in the literature.

These findings place Zn^2+^ within the broader framework of polyspermy prevention but with important distinctions. Removal of extracellular Zn^2+^ does not increase the incidence of polyspermy [18], indicating that Zn^2+^ is not required for the establishment of the canonical polyspermy blocks in *X. laevis*. Consistent with this, José et al. used *ZnT2/4 Null* mice in which fertilization-triggered zinc sparks are dramatically attenuated and found no increase in supernumerary sperm in the perivitelline space [21], and Kageyama et al. similarly report normal monospermic fertilization rates in oocyte-specific *Slc39a10* conditional knockout (cKO) mice lacking zinc sparks [22]. Notably, *Slc39a10* cKO embryos show compromised development to the blastocyst stage, likely reflecting a distinct role for intracellular zinc homeostasis during embryogenesis, separable from the extracellular Zn^2+^ signaling we describe here, highlighting that zinc operates through multiple mechanisms throughout fertilization and early development. In *X. laevis*, our data are consistent with extracellular Zn^2+^ acting primarily at fertilization rather than during subsequent development, as eggs fertilized in the absence of Zn^2+^ develop normally even when Zn^2+^ is added after fertilization. Instead, Zn^2+^ functions as a modulatory signal that enhances the robustness of fertilization control by modifying the extracellular environment and reducing fertilization competence after the initial fertilization event.

Notably, Zn^2+^-dependent remodeling of the egg extracellular structures appears to be evolutionarily conserved. In mouse eggs, the fertilization-triggered Zn^2+^ release from cortical granules establishes a transient penetration block at the zona pellucida, during which Zn^2+^ suppresses sperm motility and promotes structural remodeling of the zona to reinforce the slow block to polyspermy [20]. Concomitantly, fertilization induces a marked increase in the Zn^2+^ content of the zona pellucida, contributing to zona hardening and reinforcing the polyspermy block [20]. Recent structural and biochemical studies show that post-fertilization cleavage of zona pellucida glycoprotein 2 (ZP2) triggers its oligomerization and extensive cross-linking of zona filaments, physically hardening the egg coat and rendering it impenetrable to sperm [8]. Proposed mechanisms suggest that Zn^2+^ forms bridges between these ZP proteins that stabilize the structure, in addition to the structural changes that occur due to proteolytic reactions [20]. Together, these processes generate a progressive “penetration block,” in which components of the cortical granules gradually reduce sperm binding and penetration [42]. In our study, we utilize removal of the jelly coat with βME to demonstrate that Zn^2+^ alters the physical properties of the glycoprotein-rich structures surrounding the egg through a non-covalent mechanism, rendering them resistant to the reductive procedures that typically allow removal of the jelly coat. Separately, chelation of extracellular Zn^2+^ with ZX1 fully restores fertilization competence in both eggs and sperm, confirming that the inhibitory effects of Zn^2+^ on gamete function are dynamic and reversible. Despite differences in egg architecture, jelly coat in externally fertilizing species and zona pellucida in mammals, both systems show evidence of Zn^2+^-dependent alterations to glycoprotein-rich extracellular structures that are consistent with a role in restricting sperm entry, suggesting that the use of Zn^2+^ to modify the egg’s extracellular environment is a broadly conserved feature of fertilization across vertebrates [43].

We propose a working model in which fertilization triggers a localized, transient elevation of extracellular Zn^2+^ that acts on multiple fronts: suppressing sperm function, reducing egg competence, and stabilizing the egg’s extracellular matrix. Rather than functioning as a simple on/off switch for polyspermy prevention, Zn^2+^ serves as a modulatory signal that enhances the reliability, stability, and adaptability of fertilization control. Further study of the molecular mechanisms by which Zn^2+^ targets gametes and modifies the egg’s extracellular structures, as well as the evolutionary conservation of this modulatory role across species, will be necessary to fully define the scope of extracellular Zn^2+^ as a regulator of fertilization. By linking extracellular Zn^2+^ signaling to coordinated actions on both gametes and the egg’s extracellular environment, these findings establish Zn^2+^ as a central, previously underappreciated ionic regulator of the earliest events in embryonic development.

## Supplementary Material

Bainbridge_Supplment_Figure1_ioag161

Bainbridge_Supplment_Figure2_ioag161

Bainbridge_BOR_R2_Supplement_ioag161

## Data Availability

All data supporting the findings of this study are included within the manuscript.
