# Supplementary material for: Extracellular zinc acts as a dynamic extracellular regulator of fertilization in Xenopus laevis
Source: Biol Reprod. 2026 Jul 31;115(4):1124–33. doi: 10.1093/biolre/ioag161 (PMC13669407; doi:10.1093/biolre/ioag161)
Supplement: Supplementary file 1 [file bainbridge_bor_r2_supplement_ioag161.pdf]

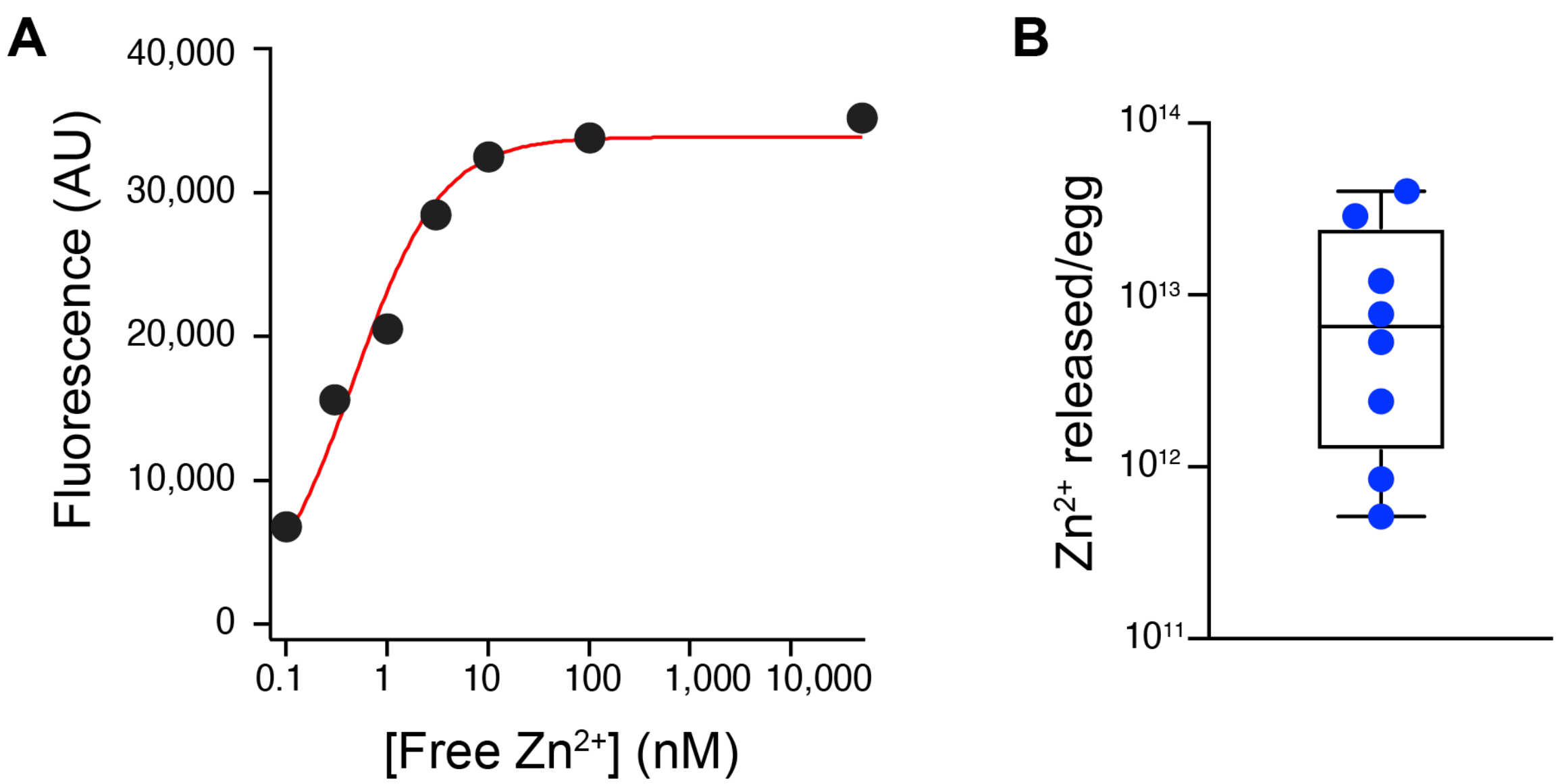

Supplemental Figure S1. FluoZin-3 standard curve and quantification of fertilization-triggered Zn^{2+} release in *Xenopus laevis*.

(A) Standard curve for FluoZin-3 tetrapotassium salt fluorescence as a function of free Zn^{2+} concentration. Free Zn^{2+} concentrations were buffered using 100 µM NTA and calculated using MaxChelator. Fluorescence intensity (RFU) is plotted as a function of labile Zn^{2+} concentration (317 pM – 3.5 µM). Data were fit with a Hill equation (solid line). (B) Distribution of Zn^{2+} ions released per egg following fertilization, quantified using the standard curve in panel A, plotted on a $\log_{10}$ scale. Each data point represents the mean value from one independent experiment (22–158 eggs per experiment; N = 8 independent experiments). The box plot shows the median and interquartile range.

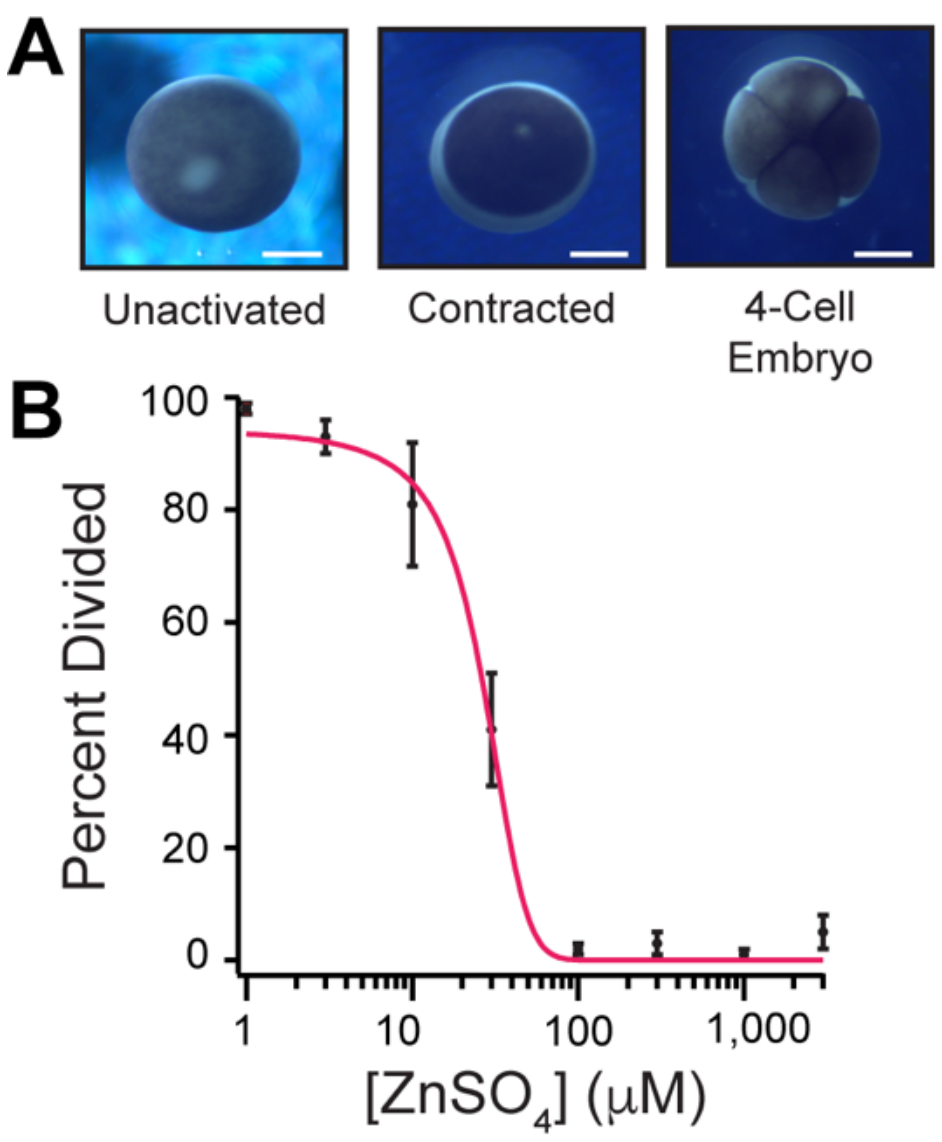

Supplemental Figure S2. Extracellular Zn^{2+} inhibits early embryonic development in a dose-dependent manner.

(A) Images of *X. laevis* eggs before sperm addition, ~15 minutes after sperm addition showing animal pole contraction, and at the 4-cell stage. Scale bar, 0.5 mm. (B) Proportion of inseminated eggs that developed cleavage furrows at the indicated concentrations of extracellular Zn^{2+}. The data were fit with a sigmoidal curve.
