# Supplementary material for: Extracellular zinc acts as a dynamic extracellular regulator of fertilization in Xenopus laevis
Source: Biol Reprod. 2026 Jul 31;115(4):1124–33. doi: 10.1093/biolre/ioag161 (PMC13669407; doi:10.1093/biolre/ioag161)
Supplement: Supplementary file 3 [file bainbridge_supplment_figure2_ioag161.pdf]

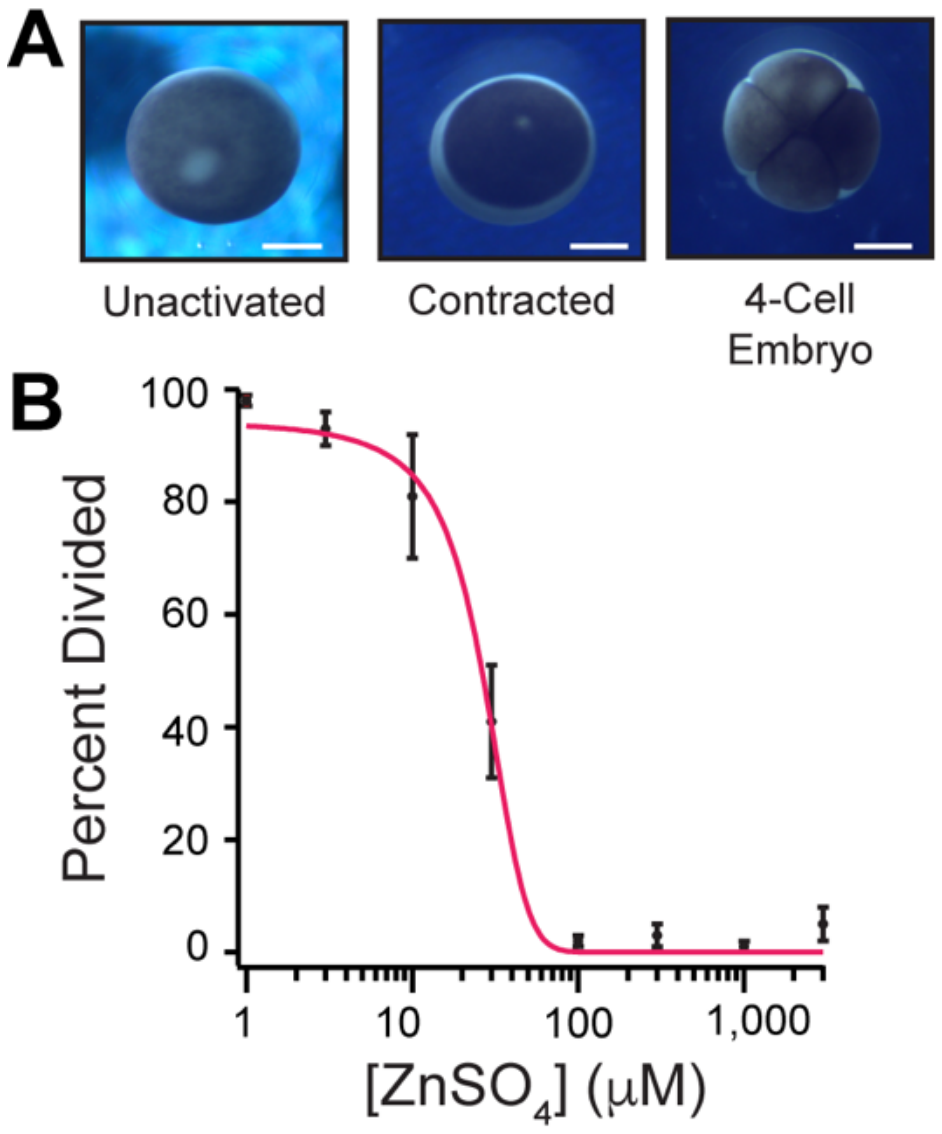

Supplemental Figure S2. Extracellular Zn^{2+} inhibits early embryonic development in a dose-dependent manner.

(A) Images of *X. laevis* eggs before sperm addition, ~15 minutes after sperm addition showing animal pole contraction, and at the 4-cell stage. Scale bar, 0.5 mm. (B) Proportion of inseminated eggs that developed cleavage furrows at the indicated concentrations of extracellular Zn^{2+}. The data were fit with a sigmoidal curve.
